# The Utilization and Diagnostic Yield of Upper Endoscopy for Evaluation of Gastrointestinal Symptoms Within the First Year After Liver Transplantation

**DOI:** 10.7759/cureus.11323

**Published:** 2020-11-04

**Authors:** Ebubekir Daglilar, Sean E Connolly, Veysel Tahan, Ari Cohen, George Therapondos

**Affiliations:** 1 Gastroenterology and Hepatology, University of Missouri-Columbia, Columbia, USA; 2 Gastroenterology and Hepatology, Ochsner Medical Center, New Orleans, USA; 3 Surgery, Ochsner Medical Center, New Orleans, USA; 4 Gastroenterology, Ochsner Medical Center, New Orleans, USA

**Keywords:** liver transplantation, endoscopy, post liver transplant gastrointestinal problems, posttransplant bleeding, posttransplant anorexia

## Abstract

Background

Gastrointestinal (GI) symptoms impact quality of life and increase health care utilization after liver transplantation (LTx). Esophagogastroduodenoscopy (EGD) is commonly used to investigate these symptoms.

Aims

The aim of this study was to investigate the diagnostic yield and utilization of EGD after LTx for common GI symptoms.

Methods

This single-center retrospective cohort study was conducted at a large liver transplant center and included all adults who underwent EGD within the first year after receiving LTx between January 1, 2015, and December 31, 2016. Biliary procedures were excluded.

Results

Of 437 patients who underwent LTx during the study period, 64 (15%) underwent EGD for the evaluation of GI symptoms within the first year of transplantation. After applying exclusion criteria, 57 (13%) cases were analyzed. GI hemorrhage (hematemesis/melena) was the most common reason (4%; n=18) for evaluation with EGD followed by nausea/anorexia (3%; n=12). Symptoms were investigated with EGD, including epigastric/abdominal pain (2%; n=9), dysphagia/odynophagia (2%; n=8), anemia (1%; n=5), diarrhea (1%; n=4), and heartburn (0.2%; n=1). The diagnostic yield of EGD was highest with GI hemorrhage (83%) followed by dysphagia/odynophagia (75%). EGD diagnostic yield was lower for the other symptoms, ranging from 0% to 25%.

Conclusions

EGD was commonly utilized within the first year of LTx, with the highest diagnostic yields for GI hemorrhage and dysphagia/odynophagia. Because of the low diagnostic yield of EGD for other symptoms, we recommend a careful selection of patients for EGD following LTx.

## Introduction

Liver transplantation (LTx) is a life-saving surgery for patients with end-stage liver disease, hepatocellular carcinoma, and other metabolic diseases of the liver [1]. Survival outcomes at one and five years posttransplantation are excellent [2], but LTx is associated with significant surgical and postsurgical complications [3]. The average age of transplant recipients has been increasing [4], and recipients often have several comorbidities including cardiac, pulmonary, renal, and gastrointestinal (GI) illnesses that can complicate postoperative recovery, decrease quality of life [5], and increase health care utilization [6]. Biliary complications, including strictures and bile leaks, are common (with an incidence of 10%-25%) but can be safely and effectively managed with endoscopic interventions [7]. Aside from biliary complications, GI symptoms include GI bleeding [8] and nausea/anorexia, which can lead to malnutrition [9] and prolonged postoperative recovery [10]. Immunosuppression following transplantation increases the risk of infectious complications that can involve the entire GI tract and lead to symptoms of dysphagia/odynophagia, epigastric/abdominal pain, and diarrhea [11]. Esophagogastroduodenoscopy (EGD) is commonly used to investigate GI symptoms. Our study aimed to determine the utilization and diagnostic yield of EGD for common GI symptoms after LTx.

## Materials and methods

Institutional Review Board approval was obtained prior to data collection. The requirement for informed consent was waived given the retrospective nature of the study. All data were collected retrospectively at a large liver transplant center (Ochsner Multi-Organ Transplant Institute, New Orleans, Louisiana, USA) from the electronic health record. All adults who underwent EGD within the first year after receiving deceased donor LTx between January 1, 2015, and December 31, 2016, were included in the study. Patients younger than 18 years of age, those who underwent biliary procedures (endoscopic ultrasound [EUS], endoscopic retrograde cholangiopancreatography [ERCP], or biliary stent removal with EGD), and living donor recipients were excluded. The following data were collected: patient age, gender, LTx date, and indications for the procedures. EGDs were performed by board-certified gastroenterologists with the American Society of Gastrointestinal Endoscopy quality metrics. Esophagitis was graded based on the Los Angeles (LA) classification system. Endoscopic findings such as LA grade A esophagitis and mild gastric/duodenal erythema with negative biopsies for *Helicobacter pylori* or viral stains were interpreted/reported as negative studies. All patients underwent EGD for the evaluation of nausea/anorexia, and biopsies from the stomach and duodenum were also obtained. All biopsies were stained for *H. pylori*, viral, or fungal stains as deemed appropriate. Descriptive analysis was used to report findings of the study.

## Results

Among the 437 patients who underwent LTx, 64 (15%) underwent EGD for the evaluation of GI symptoms within the first year of LTx. Among these, seven cases met the exclusion criteria (stent removal [n=4], variceal surveillance [n=2; EGD showed resolution of varices], follow-up of a previously known duodenal polyp [n=1]); therefore, 57 (13%) patients were included in the analysis.

Relative to the entire cohort of LTx patients, 18 (4%) patients underwent EGD for evaluation of GI hemorrhage (including hematemesis or melena). The endoscopic diagnoses were portal hypertensive gastropathy (n=4), gastric ulcer (n=4), duodenal ulcer (n=3), LA grade D esophagitis (n=1), esophageal ulcer (n=1), arteriovenous malformation (n=1), and Dieulafoy lesion (n=1). EGD did not reveal any source of bleeding in three patients.

Nausea/anorexia (3%) was the second most common reason for investigation with EGD (n=12). EGD was normal for nine patients but showed LA grade C/D esophagitis in two patients and gastric ulcer in one patient. Three (0.7%) patients who had anorexia and prolonged nausea underwent percutaneous endoscopic gastrostomy tube placement.

Epigastric/abdominal pain was investigated in nine (2%) patients, and EGD revealed mild gastric/duodenal erythema in seven patients. Two studies were normal. In the patients with mild erythema in the stomach or duodenum, biopsies obtained for pathologic examination, *H. pylori* immunohistochemical testing, and viral stains were negative in all cases.

Dysphagia/odynophagia was investigated in eight cases (2%), and EGD revealed LA grade C or D esophagitis (n=4), ischemic esophagitis (n=1), and esophageal candidiasis (n=1). Two studies were normal.

EGD was performed for the evaluation of anemia in five (1%) cases, which revealed portal hypertensive gastropathy in one case and normal in four cases. Diarrhea was the reason for EGD in four (1%) patients. Three studies were normal; one study revealed villous atrophy. One patient underwent evaluation of heartburn with normal EGD findings. EGD findings are summarized in Table 1.

**Table 1:** Summary of esophagogastroduodenoscopy findings *All biopsied and tested negative for *Helicobacter pylori* and viral inclusions. LA, Los Angeles; LTx, liver transplantation

Reason for upper endoscopy	Number of LTx recipients (n=57)	Endoscopy findings
Gastrointestinal hemorrhage (hematemesis/melena)	18	Portal hypertensive gastropathy (n=4), gastric ulcer (n=4), duodenal ulcer (n=3), LA grade D esophagitis (n=1), esophageal ulcer (n=1), arteriovenous malformation (n=1), Dieulafoy lesion (n=1), normal (n=3)
Nausea/anorexia	12	LA grade C/D esophagitis (n=2), gastric ulcer (n=1), normal (n=9)
Epigastric/abdominal pain	9	Mild gastric/duodenal erythema* (n=7), normal (n=2)
Dysphagia/odynophagia	8	LA grade C/D esophagitis (n=4), ischemic esophagitis (n=1), esophageal candidiasis (n=1), normal (n=2)
Anemia without overt bleeding	5	Portal hypertensive gastropathy (n=1), normal (n=4)
Diarrhea	4	Villous atrophy (n=1), normal (n=3)
Heartburn	1	Normal (n=1)

**Figure 1:**
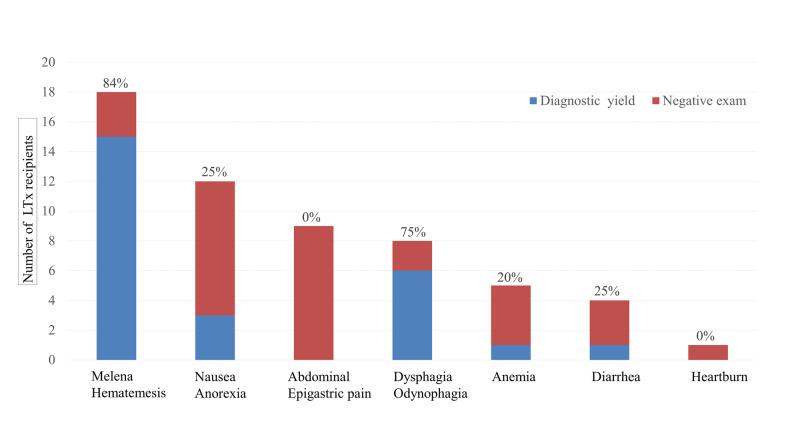
The utilization and diagnostic yield of EGD within the first year of LTx EGD, esophagogastroduodenoscopy; LTx, liver transplantation

Diagnostic yield of EGD was highest for GI hemorrhage (83%) followed by dysphagia/odynophagia (75%). EGD had lower diagnostic yields for all other symptoms (Figure 1).

## Discussion

LTx outcomes have been improving with the advances in the liver transplant field [12], but clinicians encounter frequent posttransplant-related GI problems and complications [4]. Many studies have focused on complications of surgical [13], biliary [7], infectious [11], and vascular diseases [14], but we examined the role and yield of EGD for non-biliary GI complications.

During the two-year study period, 437 consecutive deceased donor LTx recipients were included. Within the first year of LTx, 57 (13%) patients underwent EGD. Of our LTx patients, 4% underwent EGD for the evaluation of GI hemorrhage (including hematemesis or melena). No mortality was related to the GI bleeding episodes identified in our population, but they resulted in additional days of hospital stay or readmissions. The etiology of the GI bleeding in our cohort was similar to that in the non-transplant population [15], with the most common finding being peptic ulcer disease in seven cases and portal hypertensive gastropathy in four cases. Akatsu et al. reported rapid and complete resolution of portal hypertension and related complications following living donor LTx, whereas peptic ulcer disease was reported to be as high as 6.9% [16]. In our cohort, the rate of peptic ulcer disease was 2%. The high incidence of peptic ulcer disease in Akatsu et al.’s study was likely related to the use of histamine 2 receptor antagonist medications instead of proton pump inhibitors (PPIs) following LTx. In our cohort, five (1%) patients had refractory portal hypertension resulting in bleeding or anemia. In this group, EGD was performed two weeks to three months after LTx. The prevalence of post-LTx portal hypertension has been reported as high as 2.8%, resulting in variceal bleeding or ascites [17]. Doppler ultrasound evaluation is recommended for the diagnosis of portal hypertension in patients with ongoing bleeding following LTx. Hanouneh et al. reported recurrent GI bleeding masquerading as gastric variceal bleeding on EGD following living donor LTx [8], but subsequent imaging studies showed that bleeding was due to prominent submucosal gastric artery as a complication of splenic artery ligation during surgery. It is vital to differentiate these two conditions as cyanoacrylate treatment of this lesion can result in significant systemic embolization. Other causes of bleeding were severe esophagitis with LA grade D, arteriovenous malformation, and a Dieulafoy lesion. All of the bleeding cases in our cohort were effectively treated with endoscopic hemostasis techniques.

In the majority of the patients with nausea/anorexia, the underlying cause was deemed multifactorial and included postoperative narcotics use, polypharmacy, periprocedural infections, or gastroparesis. In the postoperative period, several risk factors have been proposed for the development of nausea, including anesthesia technique, duration of anesthesia, opioid administration, and patient-related factors [10]. In all 12 cases of nausea/anorexia, biopsies were obtained from the stomach or small bowel for pathological examination, but none were positive for *H. pylori* or viral inclusions. Although we did not see any viral gastritis cases, Nohr et al. reported a case of Varicella*-zoster* virus gastritis post-LTx, who also had skin manifestations and systemic involvement, and was successfully treated by antiviral agents [18].

In contrast to several studies reporting prevalence rates of *H. pylori* in patients with end-stage liver disease of 50% pretransplant and 5.6% posttransplant [16-19], we had a zero *H. pylori* infection rate in our post-LTx patients, likely because of testing and eradication of *H. pylori* prior to LTx in our patient population.

Organ transplant recipients and patients with human immunodeficiency virus (HIV ) infection are at the highest risk for infectious esophagitis, including bacterial, viral, and fungal infections that often present as dysphagia or odynophagia [20-21]. Cytomegalovirus esophagitis is the second most common GI manifestation after cytomegalovirus colitis and has been reported in 1% of the all diagnostic endoscopies [20]; in our cohort, severe esophagitis (including LA grade C/D and ischemic esophagitis) was the most common endoscopic finding for dysphagia/odynophagia followed by esophageal candidiasis. No cases of viral esophagitis (herpes simplex virus or cytomegalovirus) were seen, likely because of the effective use of prophylactic antiviral treatments. The one case of ischemic esophagitis was associated with multiple organ dysfunction, and a similar presentation was previously reported [22]. The patient with esophageal candidiasis was treated with appropriate antifungal agents, and the symptoms resolved.

In the patient with villous atrophy, testing for celiac disease was negative; therefore, the finding was attributed to the use of immunosuppressive medication (mycophenolate), as celiac-like enteropathy has been reported in several cases with the use of mycophenolate sodium [23].

Although Akatsu et al. reported an increased incidence of reflux esophagitis following LTx [16], we did not appreciate it in our study population. Our study group only had eight (2%) cases of esophagitis, which may have been the result of prophylaxis with PPIs.

Our study has limitations as it represented a single-center experience with a retrospective review of medical records. Large-scale studies are needed to understand the incidence and prevalence of the GI problems following LTx. Preliminary findings of the study were presented as a poster at the American College of Gastroenterology meeting in Orlando [24].

## Conclusions

Our study indicates that EGD has a major role in the definitive diagnosis and specific treatment of the GI problems developing after LTx. EGD diagnostic yield was low in our LTx patients with nausea/anorexia, abdominal/epigastric pain, diarrhea, and anemia. The highest EGD diagnostic yields were appreciated in LTx patients with GI hemorrhage and dysphagia/odynophagia symptoms that were managed with therapeutic interventions.
